# Changes in Parahippocampal White Matter Integrity in Amnestic Mild Cognitive Impairment: A Diffusion Tensor Imaging Study

**DOI:** 10.3233/BEN-2009-0235

**Published:** 2009-10-21

**Authors:** E. J. Rogalski, C. M. Murphy, L. deToledo-Morrell, R. C. Shah, M. E. Moseley, R. Bammer, G. T. Stebbins

**Affiliations:** ^1^Cognitive Neurology and Alzheimer’s Disease Center at Northwestern UniversityFeinberg School of MedicineChicagoILUSA; ^2^Department of Neurological SciencesRush University Medical CenterChicagoILUSA; ^3^Department of Family MedicineRush University Medical CenterChicagoILUSA; ^4^Department of Rush Alzheimer’s Disease CenterRush University Medical CenterChicagoILUSA; ^5^Department of Radiology Stanford UniversityPalo AltoCAUSA

**Keywords:** MRI, dementia, perforant path, tractography, entorhinal cortex, magnetic resonance imaging, hippocampus, memory, mesial temporal lobe, volumetry

## Abstract

In the present study, changes in the parahippocampal white matter (PWM), in the region that includes the perforant path, were investigated, in vivo, in 14 individuals with amnestic mild cognitive impairment (aMCI) compared to 14 elderly controls with no cognitive impairment (NCI). For this purpose, (1) volumetry; (2) diffusion tensor imaging (DTI) derived measures of mean diffusivity (MD) and fractional anisotropy (FA); and (3) tractography were used. In addition, regression models were utilized to examine the association of PWM measurements with memory decline. The results from this study confirm previous findings in our laboratory and others, showing that compared to controls, individuals with aMCI have PWM volume loss. In addition to volume reduction, participants with aMCI demonstrated a significant increase in MD, but no difference in FA, both in the PWM region and in fibers modeled to pass through the PWM region. Further, the DTI metric of MD was associated with declarative memory performance, suggesting it may be a sensitive marker for memory dysfunction. These results indicate that there is general tissue loss and degradation (decreased volume; increased MD) in individuals with aMCI compared to older people with normal cognitive function. However, the microstructural organization of remaining fibers, as determined by measures of anisotropic diffusion, is not significantly different from that of controls.

## 1. Introduction

Amnestic mild cognitive impairment (aMCI) refers to elderly individuals who show a predominant decline in declarative memory function but do not meet criteria for Alzheimer's disease (AD). Individuals with aMCI do show an increased risk for developing AD [36]. Therefore, studies of individuals with aMCI compared to normal controls provide a unique opportunity to learn about neuroanatomical changes associated with AD at early stages of the disease process, provide additional information on risk factors for AD, and perhaps allow for the development of earlier therapeutic interventions [1,33].

Previous *post mortem* and *in vivo* investigations of the pathophysiology of aMCI and AD have focused on examining the integrity of the hippocampus (HF) and

ISSN 0953-4180/09/$17.00 © 2009 – IOS Press and the authors. All rights reserved the entorhinal cortex (EC), since these mesial temporal lobe structures are known to be critically important for successful declarative memory [46,56]. Histopathological studies have reported a loss of entorhinal cortex layer II neurons in patients with AD and in those with MCI, compared to controls [19,23,24,30]. Consistent with the postmortem findings, quantitative *in vivo* structural neuroimaging studies have reported entorhinal and hippocampal atrophy in patients with AD and aMCI [3, 12,13,15,16,26,28,29,49]. Entorhinal cortex neurons receive multimodal sensory information from primary sensory and association cortices and relay this information to the hippocampus via the perforant path [2,23, 53]. It has been hypothesized that the memory deficits that characterize individuals with aMCI and AD may partly be a consequence of a degradation in the flow of information from the entorhinal cortex to the hippocampus [23].

Recent evidence suggests that damage to the parahippocampal white matter, in addition to atrophic changes in the hippocampus and entorhinal cortex, may contribute to memory dysfunction in aMCI and AD [27,41, 50]. Specifically, Stoub and colleagues [50] used voxel based morphometry (VBM) to demonstrate decreased white matter volume in the anterior medial portion of the parahippocampal gyrus that includes the perforant path in individuals with aMCI compared to older controls. In addition, this reduction in white matter volume was found to be a significant predictor of memory function, suggesting that white matter integrity in the region of the perforant path is important for successful memory. An important unanswered question is whether remaining white matter fibers in the perforant path region of the parahippocampal gyrus show microstructural alterations in individuals with aMCI. Degraded integrity of remaining fibers could further hinder impulse transmission and consequently contribute to the observed memory deficits in individuals with aMCI.

Diffusion tensor imaging (DTI) makes it possible to examine the microstructural integrity of white matter *in vivo* and is especially indicative for diseases causing neuronal or axonal damage [52]. This emerging technique combines MR diffusion-weighted pulse sequences with tensor mathematics to measure molecular diffusion in three dimensions. The tensor model allows for the determination of diffusion direction through calculation of the primary, secondary and tertiary eigenvalues with their associated eigenvectors. The primary direction of diffusion is determined from the primary eigenvector, and the ratio of the primary eigenvalue to secondary and tertiary eigenvalues provides a measure of anisotropy, or the directional dependence of diffusion. When considered across voxels, the primary eigenvector can be used to model the path of directional diffusion, and when used in cerebral white matter, it can provide a model of putative white matter tracts [8,11]. This methodology for modeling white matter pathways has been termed tractography.

There are several metrics that can be calculated from DTI scans, but the two most common are mean diffusivity (MD) and fractional anisotropy (FA). MD provides a measure of non-directional diffusion and is influenced by the presence of barriers to free diffusion. As barriers to free diffusion decrease (i.e., due to degradation of tissue) MD increases. In contrast to MD, FA provides a measure of directional diffusion. In intact white matter, the direction of diffusion is parallel to myelinated and unmyelinated axonal fibers, thus leading to increased FA. Damage to white matter, such as demyelination or axonal damage, decreases FA by allowing increased diffusion perpendicular to the axonal orientation. Thus, MD and FA can be used as noninvasive proxy measures of the general integrity of the tissue and the parallel organization of remaining white matter fibers respectively [35]. Previous studies have found that alterations in these metrics are present in normal aging [35,38], pathological aging (e.g. Alzheimer's disease) and several neurologic and psychiatric disorders [21,27,32,37, 42,48,54].

The primary aim of the present study was to use DTI and tractography to examine the microstructural integrity of the remaining white matter fibers in the parahippocampal gyrus in the region of the perforant path in individuals with aMCI compared to that of elderly controls with no cognitive impairment (NCI). A secondary aim was to determine the contribution of these changes to declarative memory performance.

## 2. Methods

### 2.1. Subjects

Fourteen elderly control subjects with no cognitive impairment (NCI) and 14 individuals with amnestic mild cognitive impairment participated in this study (Table 1). NCI participants were recruited from the community and aMCI participants were recruited from the Rush Alzheimer's Disease Center (RADC; Chicago, IL) clinic. Both NCI and aMCI participants were evaluated at the RADC. It is important to note that, individuals who came to the clinic with memory com-

Table 1
Demographic characteristics, MMSE and episodic memory z-scores of participants

| | NCI (n = 14) | aMCI (n = 14) |
|---|---|---|
| Women:Men | 5:9 | 10:4 |
| Age | 73.6 (+/−6.7) | 76.8 (+/−7.0) |
| Education (years) | 16 (+/−2.4) | 16.5 (+/−3.1) |
| MMSE+ | 29.4 (+/−0.8) | 26.9 (+/− 2.0)* |
| Memory z-score | 0.63 (+/− 0.30) | −0.57 (+/−0.59)* |

+Mini Mental Status Exam (MMSE): The maximum score is 30 and $\geqslant$ 27 is normal. *Significantly different from controls ($p <$ 0.005). Means and standard deviations are provided.

plaints, but did not show any cognitive impairment, were not recruited as controls. Informed consent was obtained from all participants according to the guidelines of the Institutional Review Board of Rush University Medical Center.

### 2.2. Clinical evaluation

The evaluation carried out by the RADC has been previously described [10]. Briefly, the evaluation, which was given to all participants in the study, incorporated the Consortium to Establish a Registry for Alzheimer's Disease (CERAD) [34] procedures and included a medical history, neurological examination, neuropsychological testing, informant interview and blood tests [50].

Selection as an elderly control subject required a normal neurological examination, normal cognition as determined by performance on neuropsychological tests, a Mini Mental State Examination (MMSE) score $\geqslant$ 27 [18], and age $\geqslant$ 65. A diagnosis of aMCI was given to individuals who underwent a standard clinical evaluation and were found to have an isolated deficit in the memory domain, but did not meet criteria for dementia, as previously described [50]. Exclusion criteria for entry into the study were evidence of other neurologic, psychiatric or systemic conditions that could cause cognitive impairment (e.g., stroke, alcoholism, major depression, a history of temporal lobe epilepsy), contraindication to MRI scanning (certain metal implants and cardiac pacemakers, claustrophobia), and age less than 65 years.

The episodic memory tests administered to all participants and used to define a memory deficit consisted of immediate and delayed recall of the East Boston Story [1] and of Story A from the Logical Memory of the Wechsler Memory Scale – Revised [55]. An additional test involved the learning and retention of a 10-word list from the CERAD battery [34]. The three scores for this test included Word List Memory (the total number of words immediately recalled after each of three consecutive presentations of the list), Word List Recall (the number of words recalled after a delay) and Word List Recognition (the number of words correctly recognized in a four-alternative, forced-choice format, administered after Word List Recall). Summary scores were calculated for combined performance on declarative memory tests by standardizing each of the seven memory scores. For this study, we used the means and standard deviations of each test from the baseline visits of the first wave of 86 control participants entered into an on-going longitudinal project to construct individual memory test $z$-scores for participants in the present study. For each participant, the seven $z$-scores were averaged to construct a declarative memory $z$-score.

### 2.3. Acquisition parameters and quantification of MRI data

Imaging was performed on a 1.5 T General Electric scanner (General Electric Medical Systems, Milwaukee, WI, USA) with LX Horizon high-speed gradient upgrades (Rev 11.4) at Rush University Medical Center. Foam padding and tape were used to secure participants' head to minimize movement. The scanning session was completed in one visit and consisted of a locator scan, a 3D T1-weighted spoiled gradient recalled (SPGR) scan (124 contiguous 1.6 mm thick slices acquired in the coronal plane, matrix = 256x192, field of view = 22 cm, TR/TE = 34/7msec, flip angle = 35° ), and a single-shot echo planar high resolution diffusion weighted scan (TR/TE = 12100/97ms, field of view = 25 cm, matrix = 128×128, 38 3 mm gapless slices, in-plane resolution = 1.95 mm, 2NEX, 3 repetitions). Two diffusion weights (b-values) were used: b = 0 and b = 800 s/mm$^2$. Diffusion encoding gradients were applied along a total of 24 non-collinear directions repeated six times for each slice.

SPGR scans were converted from individual slices to volumes using the DICOM toolbox in SPM 5 (Wellcome Department of Cognitive Neurology, London, UK). Post-acquisition processing of DTI images utilized an open source suite of software developed at Stanford University (http://sirl.stanford.edu/software) with modifications developed in our laboratory.

The initial processing of the DTI scans required unwarping of the eddy current distortions. Simple coregistration of the DTI images is not optimal for correcting these distortions; therefore, we utilized a correction method developed by Rohde and colleagues [40]. This algorithm combines a rigid-body 3D motion correc-

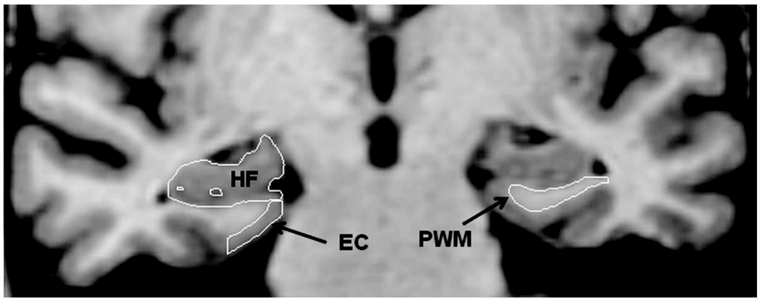

Fig. 1. A coronal slice illustrating the segmentation of the entorhinal cortex (EC), hippocampus (HF) and parahippocampal white matter (PWM).

tion (6 parameters) with a constrained non-linear warping (8 parameters) based on a model of the expected eddy-current distortions. Calculation of MD and FA proceeded from the unwarped DTI images through the calculation of the six diffusion coefficients defining the six elements of the diffusion tensor [6]. Eigenvectors, defining the three principle directions of diffusion for each voxel, and associated eigenvalues were derived from the diffusion tensor. MD and FA were derived from the eigenvalues [5,7,9]. The $b = 0$ scans were used to construct a T2-weighted image.

### *2.4. Regions of interest*

To better understand the relationship between volume loss in given mesial temporal lobe gray matter regions and white matter loss in the region of the perforant path, three regions of interest (ROI) were segmented: the parahippocampal white matter (PWM), the entorhinal cortex (EC) and the hippocampus (HF). The Analyze software package (Mayo Clinic Foundation, Rochester, MN, USA) was used for manually segmenting the ROI boundaries and for estimating the volumes of each ROI. ROIs were traced bilaterally for each subject on their high resolution T1 weighted 3D-SPGR scan. The ROI boundaries were delineated on coronal slices reformatted to be perpendicular to the long axis of the HF using two previously described and validated protocols for the EC and HF [14,20] and one new protocol for the PWM, which targets the portion of parahippocampal white matter that includes the perforant path as described below. Figure 1 shows sample tracings of all three ROIs on a single coronal MR image.

Briefly, for determining EC volume, tracing began with the section in which the gyrus ambiens, amygdala and white matter of the parahippocampal gyrus first appeared visible. The superomedial border in rostral sections was the sulcus semiannularis and in caudal sections the subiculum. The shoulder of the collateral sulcus was used as the lateral border. The latter is a somewhat conservative criterion that allowed consistency in tracings and avoided the use of different lateral borders depending on individual differences in the depth of the collateral sulcus [see for example; 25]. The lateral border was constructed by drawing a straight line from the most inferior point of the white matter to the most inferior tip of the gray matter. The last section measured was three 1.6 mm sections rostral to the image in which the lateral geniculate nucleus first appeared visible.

HF tracings started with the first section where it could be clearly differentiated from the amygdala by the alveus and included the fimbria, dentate gyrus, the hippocampus proper and the subiculum. Tracings continued on all consecutive images until the slice before the full appearance of the fornix.

Tracing of the PWM began with the slice in which the gyrus ambiens, amygdala and white matter of the parahippocampal gyrus first appear. The most caudal slice traced was one slice rostral to the first appearance of the lateral geniculate nucleus. The lateral border of the PWM was defined as the bend that signifies the junction between the parahippocampal white matter and the temporal stem. The medial border was defined as the point at which the white matter meets the gray matter of the entorhinal cortex. Tracings were carried out by ER (who was trained to be within 95% of LdeT-M). All tracings were checked, slice by slice, by LdeT-M.

To correct for individual differences in brain size, the ROI volumes (PWM, HF, and EC) were divided by total intracranial volume derived from sagittally formatted 5 mm slices (i.e., normalized). To compute intracranial volume, the inner table of the cranium was traced in consecutive sagittal sections spanning the entire brain. At the level of the foramen magnum, a straight line was drawn from the inner surface of the clivus to the occipital bone. Normalized volume for brain regions of interest was determined using the formula: absolute volume in $mm^3$/intracranial volume in $mm^3 \times 1000$.

Table 2
Total (left + right) normalized parahippocampal white matter, hippocampal, and entorhinal cortex volumes in participants with amnestic Mild Cognitive Impairment (aMCI) and healthy age-matched controls (NCI)

| | NCI (n = 14) | aMCI (n = 14) |
|---|---|---|
| Parahippocampal white matter volume | 0.754 (+/−0.13) | 0.548 (+/−0.22)* |
| Hippocampal volume | 3.77 (+/−0.41) | 3.33 (+/−0.53)* |
| Entorhinal cortex volume | 0.99 (+/−0.11) | 0.79 (+/−0.16)* |

*Significantly different from controls ($p < 0.01$). Means and standard deviations are provided.

For DTI and tractography analyses, the PWM ROIs were converted to SPM5 format for co-registration with the SPGR structural scan and the DTI scan for each participant. This conversion requires the application of the transformation matrix from the SPGR to the ROI for location alignment and re-slicing of the ROI to backfill the volume. The co-registration of the ROI to the SPGR and DTI scans utilized a rigid-body method as implemented in SPM5 which translates and rotates the SPGR volume to best match the DTI image. The co-registration parameters were applied to the PWM ROI to bring them into anatomical alignment with the DTI data. No DTI metric was shifted during the co-registration to minimize the addition of noise associated with the registration process to the DTI images. Each co-registered SPGR and ROI were manually inspected for registration error and corrected when required. The DTI values within the PWM ROIs were extracted using software developed in our laboratory. While a number of DTI scalars are available for analysis, we used the most commonly cited measures of factional anisotropy (FA) and mean diffusivity (MD).

Following DTI ROI analysis, the PWM ROIs were used as seed points to generate DTI derived tractographic models of the white matter pathways to measure the diffusion properties of the fibers passing through the PWM region. To generate the tractographic models, we used an open source suite of software developed at Stanford University (http://siri.stanford.edu/software) with modifications developed in our laboratory. These models were developed using a deterministic streamline tracking technique [8,11,31,33] with Runge-Kutta $4th$ order integration [51]. This algorithm seeks to propagate tracks based on the principal direction between voxels and on the primary eigenvector. The program assesses the degree of coherence between associated voxels in both a forward and a backward marching method. The model of the underlying white matter tracts is based on the highest directional association across voxels with a fiber termination at an FA value of less than 0.20, or an angular displacement of greater than 20 degrees. From these models, we quantified the DTI metrics of FA and MD, as well as the mean length of the modeled fibers. These values were calculated individually for each participant based on the modeled tracts propagated from the individually determined PWM ROI.

### *2.5. Statistical analyses*

Demographic differences between the two groups of participants were measured by independent $t-$tests or $\chi^2$analyses as appropriate in SPSS (SPSS, Chicago, IL). Separate independent $t-$tests were used to examine group differences in volume (PWM, EC, and HF), DTI metrics (FA and MD) of the PWM ROI and of the modeled white matter fiber tracts. Because of the number of repeated comparisons, we adopted a conservative statistical threshold of $p = 0.01$ for these analyses. Pearson correlations were performed to examine the relationship between FA and MD in our participants. Multiple regression models were used to assess the contribution of EC and HF volume loss in predicting PWM volume. Multiple regression analyses were also applied to assess the relative contribution of the *in vivo* PWM imaging measures to declarative memory performance. Statistical significance for the regression analyses was set at $p = 0.05$.

## 3. Results

Demographic data, MMSE scores and episodic memory $z$-scores are presented in Table 1. The two groups of participants did not differ in age or education, but as expected, there was a difference in MMSE [$t(26) = 4.3, p < 0.001$] scores and in episodic memory $z$-scores [$t(26) = 6.9, p < 0.001$].

Total (right + left) hippocampal, entorhinal, and parahippocampal white matter volumes were extracted for each subject, adjusted for total intracranial volume (normalized), and compared by diagnostic group.

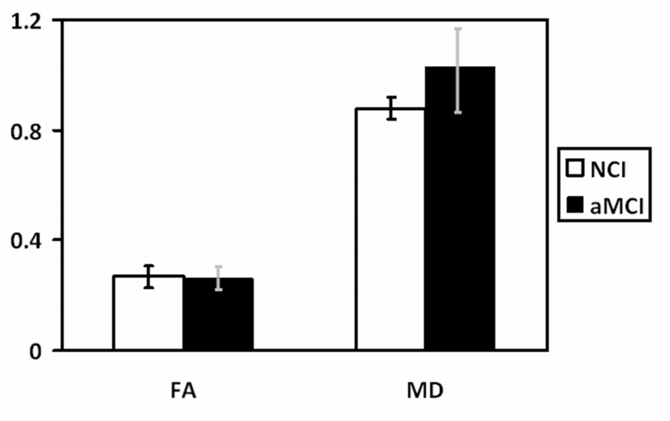

Fig. 2. Average fractional anisotropy (FA) and mean diffusivity (MD) for participants with amnestic MCI (aMCI) and healthy controls with no cognitive impairment (NCI). The vertical bars represent the standard deviation. There were significant differences between groups in MD ($p = 0.002$), but not in fractional FA ($p = 0.593$) in the parahippocampal white matter region of interest.

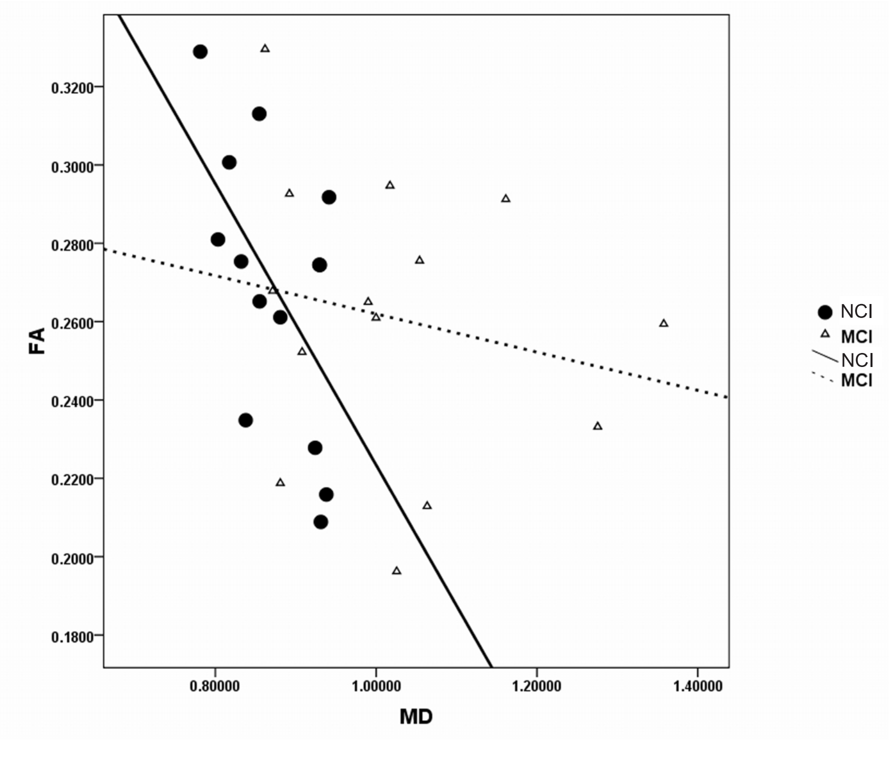

Fig. 3. Scatterplots of the parahippocampal white matter (PWM) regions of interest DTI metrics, fractional anisotropy (FA) by (MD), in amnestic mild cognitive impairment (aMCI) and controls with no cognitive impairment (NCI).

Results indicated that total volumes for each structure were significantly greater for the NCI than the aMCI group [HF: $t(26) = 2.4$, $p = 0.023$; EC: $t(26) = 3.8$, $p = 0.001$; PWM: $t(26) = 3.0$, $p < 0.006$; Table 2].

Multiple regression, with PWM volume as the outcome and EC and HF volume entered as predictors in a stepwise method revealed total normalized EC volume as the major predictor of PWM volume loss ($F[1,26]= 17.41$, $p < 0.001$), accounting for approximately 40% of the variance in PWM volume. Hippocampal volume did not enter as a predictor into the regression.

White matter integrity of remaining fibers in the parahippocampal region was measured by comparing PWM ROI values for mean FA and MD between diagnostic groups. There were no significant differences in mean FA by diagnostic group [$t(26) = 0.5$, $p = 0.593$]. However, MD [$t(26) = -3.5$, $p = 0.002$] was significantly higher in the aMCI group compared to controls (Fig. 2). These results suggest a change in general tissue structure, but not the parallel organization of remaining fibers in individuals with aMCI compared to controls.

To examine the relationship between memory performance and DTI measures of white matter integrity, a multiple regression, with memory $z$-score as the outcome and PWM ROI volume and DTI metrics (FA and MD) entered as predictors in a stepwise method, was conducted. Results revealed MD as the only significant predictor in the model (F[1,26]= 12.34, $p = 0.002$), accounting for approximately 32% of the variance in memory $z$-scores. This finding suggests that MD is the strongest predictor of memory performance.

To assess the relationship between the PWM ROI DTI metrics of FA and MD, Pearson correlations were used. Results indicated an expected negative correlation between FA and MD within the NCI group (r $= -0.57$, $p = 0.035$). However, no significant relationship between FA and MD was found in the aMCI group (r $= -0.20$, $p = 0.485$; Fig. 3), demonstrating a dissociation between the two diagnostic groups.

Up to this point, we have examined changes in the PWM ROI, but not the tractography results assessing the fibers passing through this region. We used the PWM ROIs as seed points to generate maps of fiber tracts (for an example, see Fig. 4). To examine group differences as determined by the tractography results, we assessed mean fiber length, FA and MD of the fibers passing through the PWM ROI. Measures of the integrity of the modeled white matter fibers revealed significant group differences for MD [t(26) $= -3.68$, $p = 0.001$], but not FA [t(26) $= 0.26$, $p = 0.8$; Table 3]. In addition, there were no significant group differences for mean length of fibers [t(26) $= -0.70$, $p = 0.49$; Table 3]. These FA and MD results are similar to those found in the PWM ROI analyses described above.

The relative contribution of tractographic measures (MD and FA of the fibers, and mean fiber length) to memory performance was assessed in a stepwise multiple regression. Fiber MD and FA were significant predictors of memory performance (F[1,26] = 10.36, $p = 0.001$), accounting for approximately 45% of the variance in memory $z$-scores. Mean fiber length did not enter the regression as a predictor. These results show that the relationship between FA and MD within the PWM ROI is different from that of the fibers passing through the PWM region.

Table 3
Fractional anisotropy (FA), mean diffusivity (MD) and length of the modeled white matter fiber tracts generated from the parahippocampal white matter regions of interest in participants with amnestic Mild Cognitive Impairment (aMCI) and healthy age-matched controls (NCI)

| | NCI (n = 14) | aMCI (n = 14) |
|---|---|---|
| Fiber FA | 0.35 (+/−0.03) | 0.35 (+/−0.03) |
| Fiber MD | 0.91 (+/−0.04) | 1.00 (+/−0.08)* |
| Length of fibers | 48.9 (+/−11.5) | 51.9 (+/−11.6) |

*Significantly different from controls ($p < 0.01$). Means and standard deviations are provided.

## 4. Discussion

The present study characterized, *in vivo*, white matter changes in the parahippocampal region that includes the perforant path in individuals with aMCI compared to those of elderly controls with no cognitive impairment using volumetry, DTI and tractography. The results from this study confirm and extend previous findings in our laboratory [50] and others [17], showing that compared to controls, individuals with aMCI have volume loss in the parahippocampal white matter. We also found a concomitant increase in MD in the aMCI group, for both the PWM region including the perforant path, as well as in the fibers modeled to pass through this PWM region. In addition to demonstrating group differences, regression models were used to examine the relationships between PWM changes and those of neighboring neuroanatomically connected gray matter structures (entorhinal cortex and hippocampus), as well as memory performance.

Total normalized EC, but not HF volume, was a significant predictor of total normalized PWM volume, suggesting that volume loss in the PWM may be related to cell loss in the EC. This finding has been reported by our laboratory [50] and others [43] and is consistent with the hypothesis that memory deficits that characterize individuals with aMCI and AD may partly be a consequence of a disconnection in the flow of information from the entorhinal cortex to the hippocampus via the perforant path [23,43,50].

The relationship between FA and MD in the PWM region was explored to better understand microstructural changes in white matter measured by DTI. Typically, there is an inverse relationship between FA and MD, so that as tissue is damaged or atrophied, there is an increase in MD due to increased free diffusion, but there is usually a concomitant decrease in FA due to loss of directional diffusion because of a loss of barriers to isotropic diffusion. In the region of the perforant path, the aMCI group in this study did not show the ex-

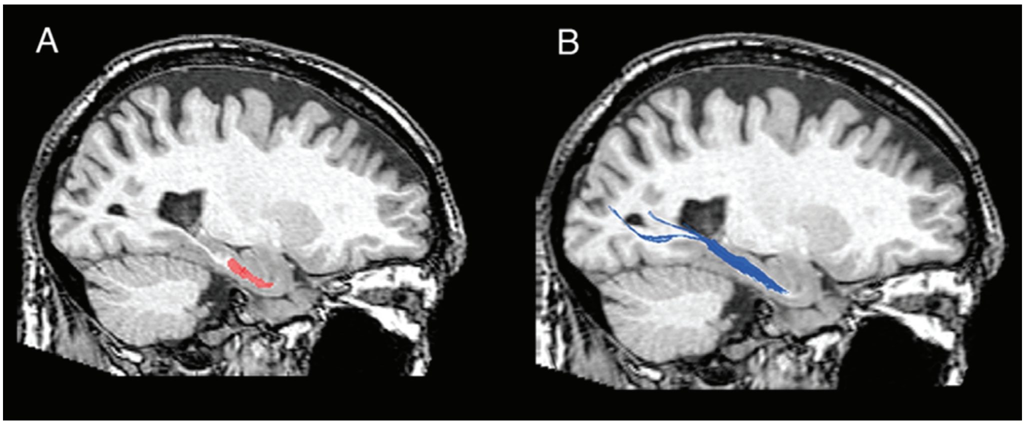

Fig. 4. Example of tractography results for an older healthy control participant. A region of interest (ROI) for parahippocampal white matter including the perforant path is drawn for each individual, based on the co-registered high resolution 3D SPGR structural scan (red ROI in panel A). This ROI is used as a seed point for the development of tractographic models of white matter pathways that pass through this ROI (blue streams in panel B). Measures of average fractional anisotropy, average mean diffusivity and average fiber length are derived from the modeled white matter pathway.

pected relationship between MD and FA values. Their MD was increased compared to the NCI group, but there was no difference in FA between the groups. This finding suggests that although individuals with aMCI have general diffusivity alterations in the PWM region, the longitudinal microstructural organization of the remaining parallel fibers of the perforant path region, as measures by FA, is comparable to that of controls. This result is in contrast to a report of decreased FA in the PWM containing the perforant path in patients with AD [43]. In that study, specific alterations in anisotropic diffusion parameters associated with myelin disruption [45] were found. This disruption of myelin function in AD may be related to increased susceptibility of PWM oligodendrocytes to metabolic and free radical formation [4].

The lack of inverse correlation between FA and MD, while less common, has been reported in individuals with MCI [17], as well as in studies of patients with HIV [48] and children with 22q deletion syndrome [44]. Unfortunately, the mechanism for tissue disruption cannot be definitively determined using DTI. The neurodegenerative changes taking place in this region are exceedingly complex and the changes in MD may reflect a combination of processes including loss of axons, dendrites and tissue degradation due to anterograde Wallerian degeneration. This may be a consequence of the cell loss in layer II of the EC and is consistent with the EC volume loss reported here and by others [43,50]. Controlled pathological studies will be important for disentangling the temporal course and mechanisms of the neurodegeneration.

A second aim of the study was to examine the contribution of the *in vivo* imaging measures from the PWM ROI to memory performance. Results from the ROI analyses revealed that MD in the white matter region containing the perforant path was the only significant predictor of memory performance, demonstrating the functional relevance of the diffusivity changes. Our results also suggest that the memory deficits that characterize individuals with aMCI do not require PWM region changes in FA. It is possible that FA may play a greater role in predicting memory performance as the disease progresses to meet the criteria for clinical AD. Future cross-sectional and longitudinal studies, comparing the PWM region integrity between the three diagnostic groups (NCI, aMCI and AD), are required to characterize the temporal changes in the perforant path region.

The tractography results indicated that both FA and MD of the fiber tracts generated from the PWM ROI contributed significantly to memory performance in the regression models. The significant addition fiber FA to the prediction of memory performance suggests the relationship between the fibers in the local PWM ROI and the fibers that pass through the PWM region is different. One caveat of tractography estimates from a single ROI seed point is that it only provides information about the fibers passing through this region and is not limited to fibers originating in the PWM region that includes the perforant path. Despite this limitation, it is important to note that the diffusion data of the tracked fibers corroborate the PWM ROI data, which show a consistent increase in MD, but no change in FA.

Though several studies have examined white matter changes in MCI and AD, few have targeted the perforant path as a region of interest using DTI [27,39, 41,43,47]. The results from these studies have been mixed. The inconsistent findings may be attributed to the methodological variability between studies, including different approaches to the acquisition and analysis of DTI parameters (e.g., diffusion weights, eddy current correction), inconsistency in the DTI metrics reported (e.g., coherence index, FA, MD, axial and radial diffusivity measures) [17,22,27], and variability in the region of interest definitions (e.g. spherical, rectangular) [17,22].

The diversity of methodologies used in previous studies limits generalizability and makes it difficult to compare the previous results to the present findings. For example, Kalus and colleagues [27] manually traced the perforant path ROI but only examined the coherence index measure of DTI, leaving out more common measures of FA or MD from the analysis, thus preventing parallel comparison to the findings in the present study. The temporal lobe white matter DTI results from Fellgiebel and colleagues [17] are consistent with those reported in this study, i.e., increased MD but no change in FA. However, their method of ROI placement (rectangular) was not as anatomically specific as our targeted manually traced ROI. The present study applied comprehensive eddy current corrections to the DTI images, used a clearly defined, anatomically driven protocol for segmenting the PWM region and reports data using the two most common DTI metrics, MD and FA. These methodological considerations ensured accurate representation of the white matter region and will allow for replication of the study by future investigators.

In summary, the present study used *in vivo* imaging to demonstrate regional tissue loss and degradation as evidenced by decreased volume and increased MD within the PWM region that includes the perforant path in individuals with aMCI compared to cognitively intact elderly controls. However, the parallel microstructural organization (as measure by FA) of the remaining fibers within the PWM region was similar between diagnostic groups. Further, it is not surprising that the DTI metric of MD, but not FA, was predictive of memory performance since FA was not significantly different between the aMCI and control groups. Taken together, these findings suggest that even with intact microstructural organization, tissue loss and degradation of the perforant path fibers contribute to the memory loss that characterizes individuals with aMCI who are at risk for developing AD. Though the exact mechanism cannot be determined, our data are in line with the hypothesis that parahippocampal white matter degradation causes a disruption of multimodal input from the entorhinal cortex to the hippocampus. It is important that future research characterizes the temporal changes in the microstructural organization of the PWM throughout the neurodegenerative process from aMCI to clinical AD.

## Acknowledgments

The research reported here was supported by grants P01 AG09466 and P30 AG10161 from the national Institute on Aging, National Institutes of Health. Dr. Rogalski received support from a Postdoctoral Training Grant (T32 AG00257) from the National Institute on Aging.
